# Loss of the *Arabidopsis thaliana* P_4_-ATPase ALA3 Reduces Adaptability to Temperature Stresses and Impairs Vegetative, Pollen, and Ovule Development

**DOI:** 10.1371/journal.pone.0062577

**Published:** 2013-05-07

**Authors:** Stephen C. McDowell, Rosa L. López-Marqués, Lisbeth R. Poulsen, Michael G. Palmgren, Jeffrey F. Harper

**Affiliations:** 1 Department of Biochemistry and Molecular Biology, University of Nevada, Reno, Nevada, United States of America; 2 Department of Plant Biology and Biotechnology, Centre for Membrane Pumps in Cells and Disease (PUMPKIN), University of Copenhagen, Danish National Research Foundation, Frederiksberg, Denmark; Institute of Botany, Chinese Academy of Sciences, China

## Abstract

Members of the P_4_ subfamily of P-type ATPases are thought to help create asymmetry in lipid bilayers by flipping specific lipids between the leaflets of a membrane. This asymmetry is believed to be central to the formation of vesicles in the secretory and endocytic pathways. In *Arabidopsis thaliana*, a P_4_-ATPase associated with the *trans*-Golgi network (ALA3) was previously reported to be important for vegetative growth and reproductive success. Here we show that multiple phenotypes for *ala3* knockouts are sensitive to growth conditions. For example, *ala3* rosette size was observed to be dependent upon both temperature and soil, and varied between 40% and 80% that of wild-type under different conditions. We also demonstrate that *ala3* mutants have reduced fecundity resulting from a combination of decreased ovule production and pollen tube growth defects. *In-vitro* pollen tube growth assays showed that *ala3* pollen germinated ∼2 h slower than wild-type and had approximately 2-fold reductions in both maximal growth rate and overall length. In genetic crosses under conditions of hot days and cold nights, pollen fitness was reduced by at least 90-fold; from ∼18% transmission efficiency (unstressed) to less than 0.2% (stressed). Together, these results support a model in which ALA3 functions to modify endomembranes in multiple cell types, enabling structural changes, or signaling functions that are critical in plants for normal development and adaptation to varied growth environments.

## Introduction

Cellular membranes are constantly changing, with the addition and removal of lipids and proteins. Eukaryotes utilize two different types of ATP-dependent enzymes to reorient lipids within membranes; flippases (P_4_ subfamily of P-type ATPases) and floppases (ABC transporters) [1]–[3]. In many situations, lipids can also be translocated by a scramblase that functions without a direct link to ATP hydrolysis. In the case of P-type ATPases, ATP hydrolysis involves a phospho-aspartate intermediate, the formation and degradation of which during the catalytic cycle is coupled to conformational changes in the transmembrane domain. Of the five subfamilies of P-type ATPases [4]–[8], members of the P_4_ subfamily have only been identified in eukaryotes [7]. While P-type ATPases are well studied in the context of translocating different ions across membranes, including Na^+^/K^+^, H^+^, Ca^2+^, and heavy metals [8], very little is known about the mechanism and function of P_4_-ATPases.

Evidence indicates that P_4_-ATPases flip specific phospholipids from one membrane leaflet to the other [9], [10]. In yeast, there is evidence for the transport of phosphatidylserine (PS), phosphatidylethanolamine (PE) and phosphatidylcholine (PC) by two P_4_-ATPases that reside primarily in the post-Golgi network (Drs2p and Dnf3p) [11] as well as two P_4_-ATPases located in the plasma membrane (Dfn1p and Dnf2p) [12]. The proposed lipid asymmetry generated by flippases plays an important role in membrane trafficking, either in facilitating the formation of membrane curvature in vesicle budding, or through regulation of surface features involved in signaling and targeting [1], [3], [13]–[17]. Defects in vesicular trafficking have been reported for P_4_-ATPase mutants of yeast [12], [18]–[21], plants [22], and animals [1].

In *Arabidopsis thaliana*, twelve P_4_-ATPase proteins have been identified: Aminophospholipid ATPase 1 (ALA1) to ALA12 [4]–[6]. Isoforms ALA2 and ALA3 have been shown to provide flippase activity when co-expressed with a beta-subunit in a yeast mutant deficient for its endogenous PM localized P_4_-ATPases (*dnf1Δdnf2Δ*) [22]–[24]. In plants and yeast, P_4_-ATPases are known to have different substrate specificities. For example, ALA2 specifically transports PS [24] while ALA3 transports PE as well as PC and PS to a lesser degree [22]. Evidence suggests that ALA1 functions at the PM [25], and is important for cold tolerance [23]. For ALA3, Poulsen et al. [22] provided evidence that this protein localizes to the *trans*-Golgi network, and its loss results in impaired root and shoot growth. In *ala3* loss of function mutants, the length of primary roots is reduced by ∼3-fold and root caps fail to release border cells. Root columella cells of *ala3* plants appeared to lack a type of *trans*-Golgi network-derived secretory vesicles loaded with mucilage [22]. Zhang and Oppenheimer [26] also reported aberrant trichome expansion, increased root hair length, and pollen defects that resulted in a segregation distortion [26]. However, this study failed to confirm the small rosette phenotype noted by Poulsen et al. [22] and suggested that this phenotype should be re-evaluated.

In this work, we provide evidence that the root, rosette and reproductive phenotypes for *ala3* knockouts are strongly dependent upon growth conditions. In addition, we show that *ala3* knockouts grown under optimal conditions have a reduced fecundity that is linked to both ovule production and pollen fitness. *In-vitro* pollen growth assays and seed set patterns in *ala3* siliques indicate that mutant pollen tubes grow slower and achieve less overall length than wild-type. Furthermore, cytoplasmic streaming appears less organized in growing *ala3* pollen tubes than in wild-type. These results support a model in which ALA3 activity modifies membranes in multiple cell types and is critical to plants for both normal development and the ability to cope with different growth environments.

## Results

### ALA3 Gene Disruptions

**Figure 1:**
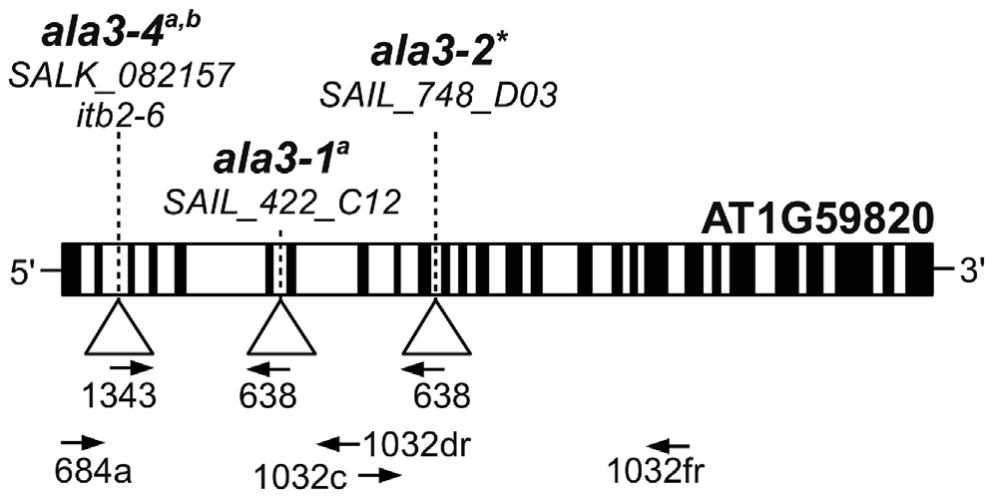
Diagram of ALA3 showing T-DNA disruptions. Filled boxes represent exons and open boxes represent introns. T-DNA insertions are represented with triangles and identified by *ala3* allele numbers, allele accessions and *itb* allele numbers where appropriate. Arrows identify oligos used for PCR genotyping and point in the 5′ to 3′ direction. The primers corresponding to the T-DNA left-borders are 1343 (SALK) and 638 (SAIL). The left border junction of *ala3-2* is: CTTGTGAATTATTAACTCCTGCTTCGAcaacttaataacacattgcggacg. Capital letters represent *ALA3* DNA and lowercase letters represent T-DNA. *Isolated in this study. ^a^Published by Poulsen et al. [22]. ^b^Published by Zhang and Oppenheimer [26].

Three independent *ala3* alleles were used in this study (Figure 1). The *ala3-1* (SAIL_422_C12) and *ala3-4* (SALK_082157) alleles were previously used by Poulsen et al. [22] and shown to have stunted growth phenotypes for roots and rosettes. The *ala3–4* allele corresponds to the *itb2–6* allele used by Zhang and Oppenheimer [26]. Here we expanded our analyses to include a third independent allele, *ala3-2* (SAIL_748_D03). All three lines were backcrossed multiple times to minimize the presence of second site mutations. Lines used for *ala3-1* and *ala3-2* were shown to be segregating a single basta-resistance marker, which is encoded within the T-DNA (Table 1, female outcrosses). Expression of *ALA3* transgenes have been shown to rescue the *ala3* root, rosette [22] and trichome [26] phenotypes, providing evidence that the phenotypes are due to a loss or low levels of ALA3 expression.

**Table 1:** Segregation analysis of *ala3* indicates a temperature-sensitive defect in transmission through the male gametophyte.

♂×♀	Cross Description	Stress	Assay	Expected (%)	Observed (%)	n	p-Value
*ala3(-1, -2, -4)(+/−)* ×Same	Selfed	−	*ala3(−/−)*	25^a^	9.4, 9.7, 9.2	636, 290, 195	All <0.0001
*ala3(-1, -2, -4)(+/−)* ×Same	Selfed	+	*ala3(−/−)*	∼9.5^b^	0.8, 1.6, 0.8	367, 516, 133	All <0.0001
*ala3(-1, -2, -4)(+/−)* ×WT	Male Outcross	−	*ala3(-)*	50^a^	16.9, 6.9, 19.0	534, 245, 426	All <0.0001
*ala3(-1, -4)(+/−)* ×WT	Male Outcross	+	*ala3(-)*	16.9-19.0^b^	0, 0	236, 254	All <0.0001
WT x *ala3(-1, -2, -4)(+/−)*	Female Outcross	−	*ala3(-)*	50^a^	46.6, 42.7, 41.5	361, 218, 176	0.18, 0.03, 0.02

Under unstressed conditions, the observed results were compared to an expected Mendelian segregation. Results of assays performed under hot-day/cold-night temperature stress conditions (Figure S2) were compared to the results of the same assay performed under unstressed conditions. Statistical significance was determined by the Pearson’s Chi-Squared test.

a Expected percentages based on Mendelian segregation.

b Expected percentages based on unstressed results.

### The *ala3* Rosette Size Reduction Varies with both Temperature and Soil Conditions

**Figure 2:**
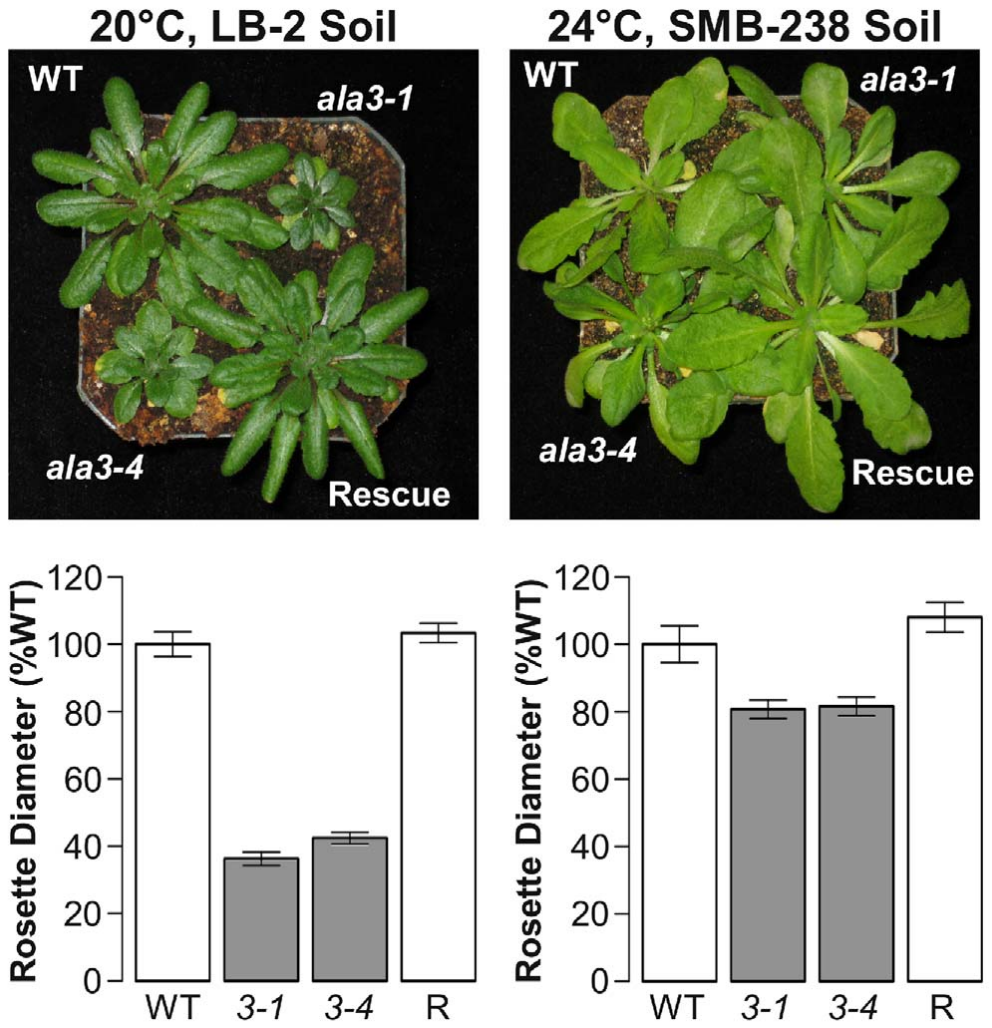
The size of *ala3* rosettes relative to wild-type varies with growth conditions. Representative examples and quantitative analysis of strong (left) and weak (right) presentations of the *ala3* rosette size phenotype. The growth conditions shown in the panels on the left are the same as those used by Poulsen et al. [22] to report the reduced rosette size phenotype of *ala3* mutants. Rosette size was measured at the time of bolting as the average length of the three longest rosette leaves. Rosette sizes were normalized to the wild-type mean and are reported as mean ± SE. Genotypes significantly different from wild-type (p<0.05, Welch’s t-test) appear in gray. Column label abbreviations are as follows: WT represents the wild-type controls; 3-1 and 3-4 represent *ala3-1* and *ala3-4* mutants, respectively; and R represents *ala3* plants rescued by the expression of full length ALA3. Representative results are shown for three independent experiments, n = 7–9 plants for each genotype/condition combination.

To further investigate the reproducibility of the reduced rosette size phenotype [22] [26], *ala3* and wild-type plants were grown in parallel under four different combinations of soil (SMB-238 and LB-2) and temperature (20°C and 24°C) conditions. Rosette sizes were measured at the time of bolting as the average length of the three longest rosette leaves. We observed a reduction in *ala3* rosette size that varied independently with both temperature and soil between 40% (20°C, LB-2 soil) and 80% (24°C, SMB-238 soil) that of wild-type (Figure 2, Figure S1). This condition-dependent variation in the *ala3* rosette size phenotype provides a possible explanation for the discrepancy between the results of Zhang and Oppenheimer [26] and Poulsen et al. [22]. Nevertheless, the average size of *ala3* rosettes was significantly smaller than that of wild-type under all conditions tested (p<0.05, Welch’s t-test).

### The *ala3* Root Length Reduction Varies with both Temperature and Growth Media

**Figure 3:**
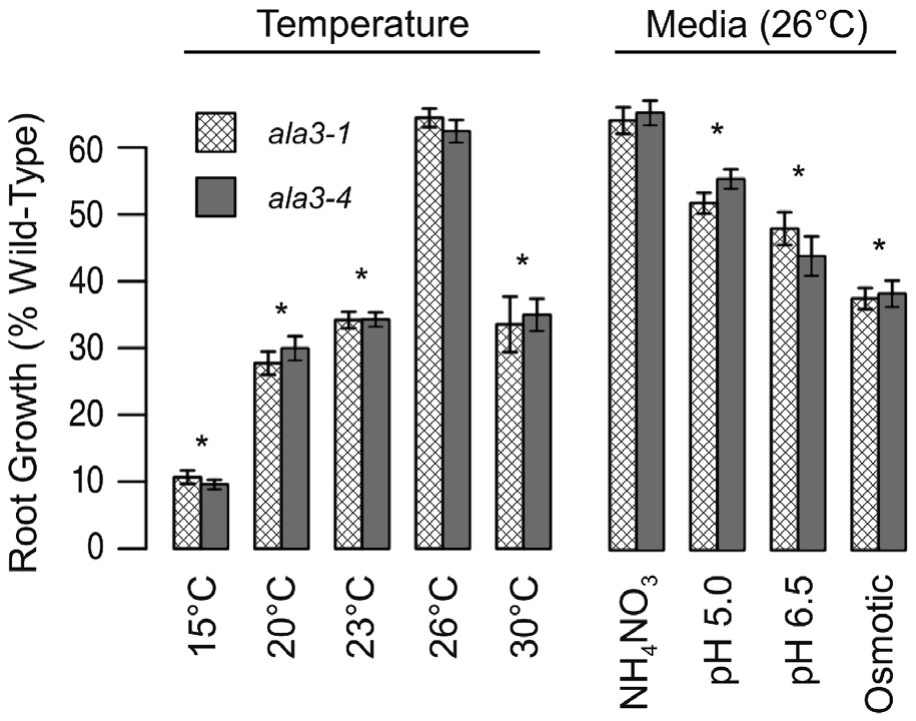
The length of *ala3* roots relative to wild-type varies with growth conditions. Seedlings were grown under 24 h fluorescent light on ½×MS media until the longest roots reached the bottom of the plate (∼7 cm). The column labels represent the conditions used in the assays. For experiments testing variations in growth media, plants were all grown at 26°C and media was amended with either 15 mM NH_4_NO_3_ (pH 5.7), KOH to adjust media to pH 5.0 or 6.5, or 4.5% sucrose to create an osmotic challenge. Root lengths were normalized to the wild-type mean and average results (±SE) for three independent experiments (n≥19 for all conditions except: n = 9 for 15°C, and n = 6 for 30°C) are presented for *ala3-1* (crosshatched bars) and *ala3-4* (filled bars). * Significantly different from *ala3* root growth at 26° on unmodified media (p<0.05, Welch’s t-test).

To determine if the *ala3* root growth phenotype [22], [26] also varies with growth conditions, wild-type and *ala3* seedlings were germinated and grown at different temperatures or on modified media. The reduction in *ala3* root growth was observed to be strongly dependent upon temperature (Figure 3). The phenotype was the least pronounced at 26°C, with *ala3* roots growing 63% as long as wild-type. However, at 30°C or 15°C, *ala3* roots were 34% and 10% as long as wild-type, respectively. Prolonged growth at 15°C was lethal to *ala3* seedlings, as they did not recover after being returned to 23°C, whereas the wild-type controls were all viable. Conditions other than temperature were also found to exacerbate the root growth phenotype (Figure 3). An additional 10–20% reduction in relative root length was observed for high pH (pH 6.5), low pH (pH 5.0), and high osmolarity (4.5% sucrose).

### The Fitness of *ala3* Pollen Is Further Reduced by Hot/Cold Temperature Stress

To determine if the *ala3* pollen transmission defect observed by Zhang and Oppenheimer [26] was also dependent on growth conditions, the transmission of the *ala3* allele in heterozygous plants was observed under standard temperature conditions (20–22°C) and under a temperature stress that cycled between hot-days (40°C peak) and cold-nights (−1°C low) (Figure S2) (Table 1). In agreement with Zhang and Oppenheimer [26], outcrosses performed under standard conditions demonstrated that the transmission of *ala3* through pollen was reduced to 6.9–19%, representing a ∼3-fold decrease from the expected 50%. This baseline segregation distortion was exacerbated by more than 90-fold in plants that were pollinated at unstressed temperatures and then immediately moved to the hot-day/cold-night stress regime. In pollen outcrosses yielding 490 progeny, no transmission of the *ala3* allele was observed. Similarly, temperature stress reduced the frequency of homozygous mutant progeny in self-fertilized *ala3 (+/−)* plants from ∼10% (unstressed) to ∼1% (stressed).

### *ala3* Pollen Tubes Are Slow and Short

**Figure 4:**
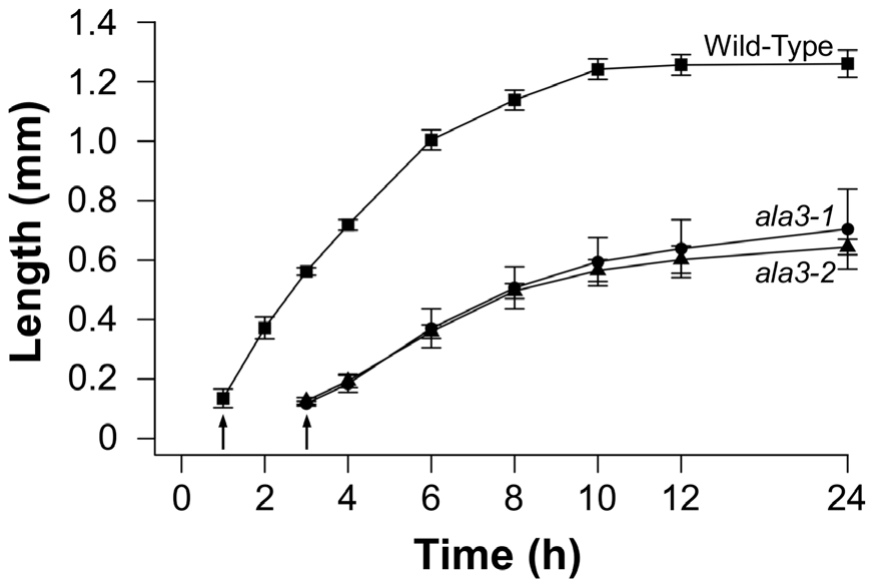
*In-vitro* assays show *ala3* pollen tubes have delayed emergence, slow growth and shorter overall length. Pollen was placed on pistils, either from the corresponding genotype or from surrogate *ms-1* plants, and the pistils were placed on pollen tube growth media. Pollen tubes growing out of the pistils were measured over a 24 h time course. Arrows represent time when buds were first observed. Lengths were reported for each time point as the average length of the 10 longest pollen tubes. Values and error bars represent the mean ± SE of three independent experiments for wild-type and *ala3-1*, and two independent experiments for *ala3-2*.

To quantify growth defects associated with *ala3* pollen, *in-vitro* growth assays were done with two independent alleles, *ala3-1* and *ala3-2*, over a 24 h time course (Figure 4). With the *in-vitro* growth conditions used here (which included a stigma to promote germination), the *ala3* pollen began to germinate approximately 2 h after wild-type. After a 24 h growth period, the overall length of *ala3* pollen tubes was about 2-fold less than wild-type. At the point of maximum pollen tube growth rates, *ala3* tubes were 2-fold slower than wild-type.

To evaluate the *in-vivo* relevance of *ala3* pollen tube growth defects, pollen from *ala3 (+/−)* plants was used to fertilize wild-type pistils and the resulting mature siliques were divided into three sectors of equal length (top, middle and bottom). Without growth defects, *ala3* could be expected to transmit to all three sectors equally, with 33% of the total transmission in each sector. However, 74%–100% of *ala3* pollen transmission was observed in the top sector, whereas no transmission of *ala3* was observed in the bottom sector (Table 2). These results indicate that the competitive fitness of *ala3* pollen relative to wild-type decreases in the distal region of the pistil, consistent with *in-vitro* growth assays showing *ala3* pollen tubes to be slow and short (Figure 4).

**Table 2:** The transmission of *ala3* through pollen is restricted to the top 2/3 of the silique.

		% Total *ala3* Transmission				
♂×♀	Assay	Top	Middle	Bottom	n	p-Value
Expected	n/a	33	33	33	n/a	n/a
*ala3-1(+/−)*×WT	*ala3(-)*	93	7	0	41	<0.0001
*ala3-2(+/−)*×WT	*ala3(-)*	100	0	0	17	<0.0001
*ala3-4(+/−)*×WT	*ala3(-)*	74	26	0	39	<0.0001

Wild-type and *ms-1* pistils were fertilized with *ala3 (+/−)* pollen and the resulting siliques were divided into three sectors of equal length: Top (stigma end), Middle, and Bottom (base of the silique). The observed results are compared to an expected equal distribution of mutant alleles across all three sectors. Statistical significance was determined by the Pearson’s Chi-Squared test.

### Fecundity of *ala3* Mutants is Reduced Primarily by Pollen Defects and Reduced Ovule Abundance

**Figure 5:**
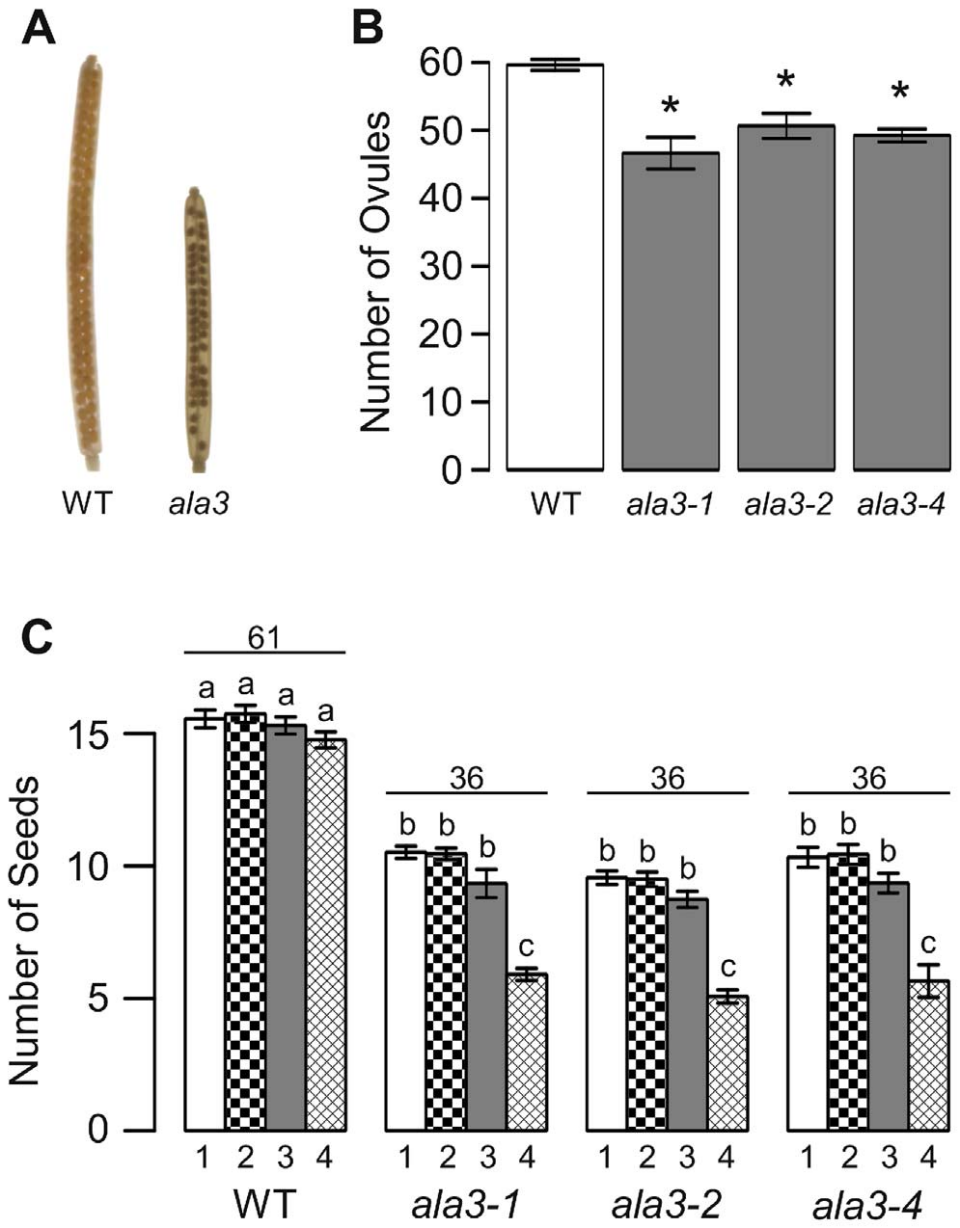
Loss of *ALA3* results in reduced seed set with an uneven distribution of seed. (A) Representative examples of wild-type and *ala3* siliques cleared with 70% EtOH to show seed positions. (B) Ovule number is reduced in *ala3* pistils, but not sufficiently to account for the total reduction in seed set. Average results (±SE) are reported for two independent experiments, n = 14–18 pistils for *ala3* mutants and n = 38 pistils for Col-0. Pistils were collected from a total of 5–6 different plants for each genotype. (C) Graph of seed set by sector. Siliques were divided into four sectors of equal length, with sector 1 at the top (stigma end) of the silique and sector 4 at the base of the silique. Average results (±SE) are reported for two independent experiments, n = 30–36 siliques. Siliques were collected from a total of 6–7 different plants for each genotype. Sector numbers appear below each column and the average total seed set for each genotype is given above the corresponding sector data. *Significantly different from wild-type (p<0.05, Welch’s t-test). ^a,b,c^Columns sharing common labels (letters) are not significantly different from each other (p>0.05).

In homozygous *ala3* mutants, seed set in each silique was decreased to ∼59% that of wild-type (Figure 5). A high frequency of empty seed positions were observed within *ala3* siliques, the majority of which were near the bottom of the silique (Figure 5a, c). This uneven seed distribution was reversed by manual fertilization of *ala3* pistils with wild-type pollen (Figure S3), consistent with the potential that homozygous plants either shed less pollen or have defective pollen. However, an explanation based on a pollen fitness problem is favored by *in-vitro* growth (Figure 4) and *in-vivo* competition (Table 2) assays, both of which indicate a pollen defect.

To evaluate whether female reproductive defects also contribute to *ala3* seed set reduction, the number of ovules in *ala3* pistils were counted and female outcrosses were performed. The number of ovules in *ala3* pistils was found to be reduced to 78–85% that of wild-type controls (Figure 5b). In addition, a variable female-side transmission deficiency was observed. In the case of *ala3-2 and ala3-4*, the transmission was reduced from an expected 50% to ∼42% (p<0.05, Pearson’s Chi-Squared test) (Table 1). However, the third allele (*ala3-1*) provided ambiguous results, as it was neither significantly different from an expected 50% transmission, nor significantly better than the ∼42% transmission observed for the other two alleles. The potential impact of different growth conditions on ovule number or penetrance of a female gametophytic deficiency was not evaluated. Nevertheless, under standard growth conditions, a pollen fitness deficiency and reduced ovule number appear to be the most significant contributions to the reduced seed set in *ala3* siliques.

### Cytoplasmic Streaming is Disorganized Near the Tip of *ala3* Pollen Tubes

**Figure 6:**
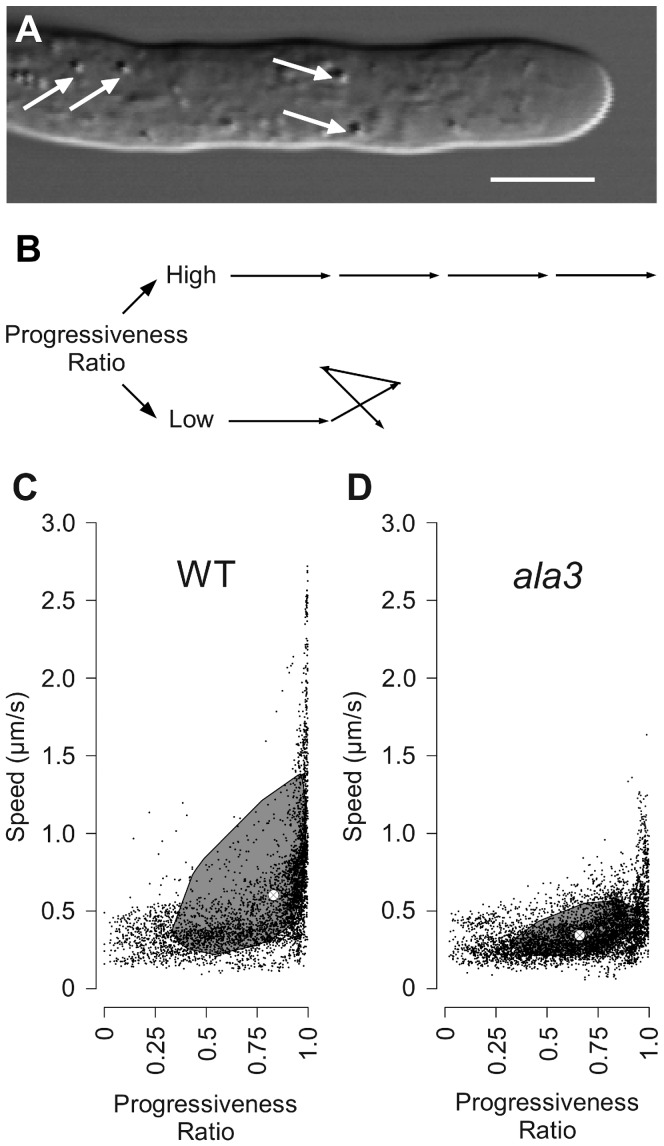
Vesicular speeds and progressiveness ratios show that vesicular movement is altered in *ala3* pollen tubes. (A) Wild-type pollen tube with white arrows pointing to representative vesicles visible with DIC optics. Scale bar = 5 µM. (B) Exemplary model trajectories with high and low progressiveness ratios. (C and D) Bagplots [29] of vesicular speed and progressiveness ratios for vesicles within (C) wild-type and (D) *ala3* pollen tubes. The shaded region represents an area containing 50% of the data points. The two-dimensional median is represented by the crosshairs within the shaded region. N = 7 for *ala3* and n = 8 for wild-type.

As a first step in evaluating *ala3* pollen for cellular deficiencies, growing pollen tubes were analyzed for changes in cytoplasmic streaming. Using DIC microscopy, the movements of organelles and large vesicular bodies were followed for 3–4 s time periods, with images taken at regular intervals of ∼0.75 s (Figure 6, Movie S1, and Movie S2). A visual inspection of the supplemental movie files suggests that streaming in mutant *ala3* pollen tubes (n = 7) is less organized than wild-type (n = 8).

To quantitatively describe vesicular behavior, we calculated the average speed and progressiveness ratio of each vesicle for which data was collected. Briefly, the progressiveness ratio is a measure of the straightness of a trajectory [27], [28], (see Equation 1 and Figure 6b). The speeds and progressiveness ratios for vesicles within wild-type and *ala3* pollen tubes are shown as bagplots [29] in Figures 6c and 6d, respectively. On average, vesicles in *ala3* pollen tubes were ∼2-fold slower, (WT, 0.60 µm/s; *ala3*, 0.35 µm/s; p<0.01 Welch’s t-test), and showed a ∼20% decrease in progressiveness ratio (WT, 0.83; *ala3*, 0.66; p<0.01 Welch’s t-test) relative to wild-type.

### Loss of ALA3 does not Affect the Lipid Composition of Pollen

**Figure 7:**
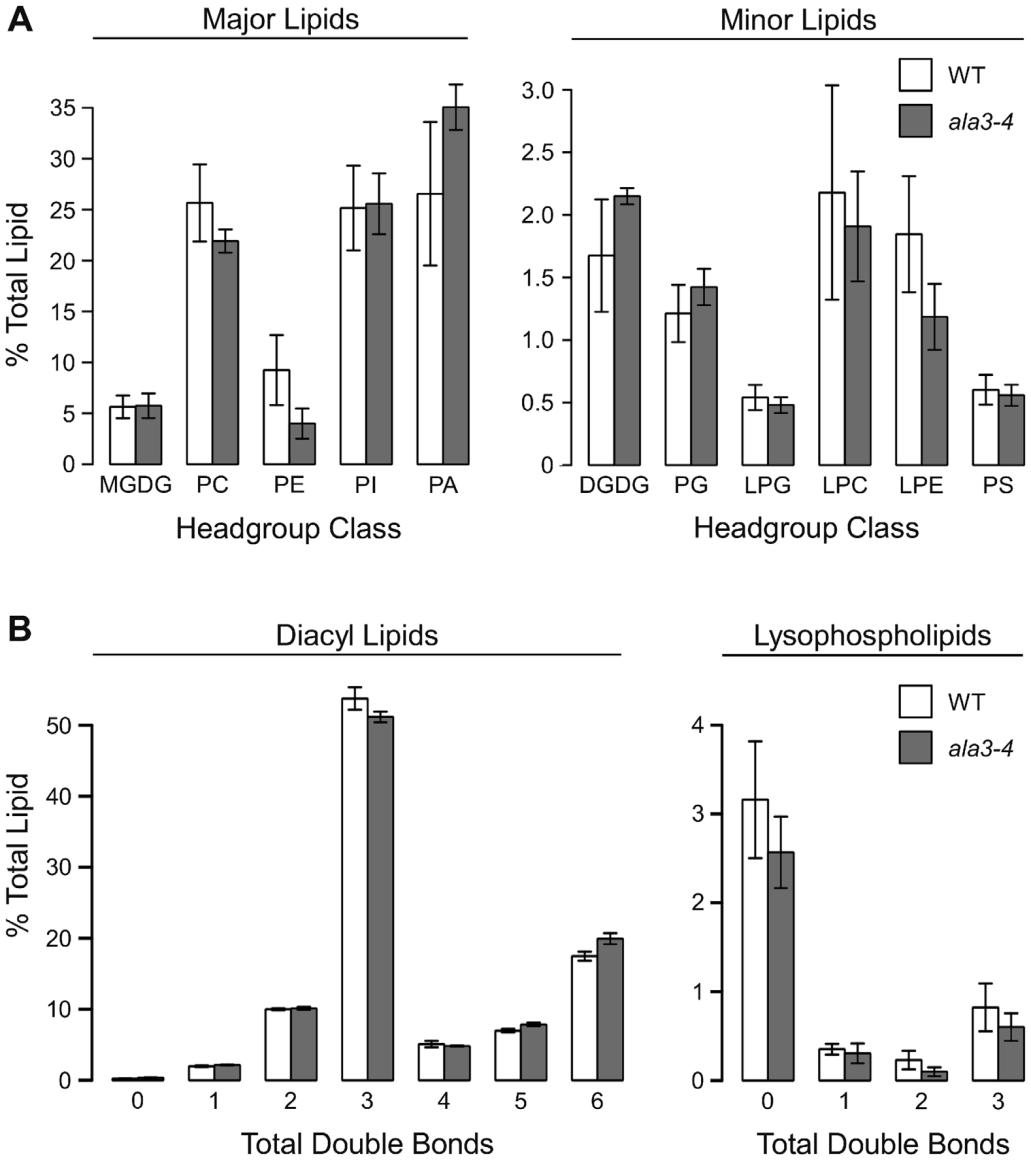
The lipid composition of *ala3* pollen is similar to wild-type. Lipid concentrations were measured using tandem mass spectrometry (MS/MS) that detected 11 different head-groups and quantified the acyl carbons and double bonds within the corresponding acyl side chain(s). Concentrations are expressed as a percentage of the total lipid detected for a specific sample and are represented as mean ± SE. Pollen was collected from independent groups (n = 4 for WT and n = 3 for *ala3-4*) of ∼75 plants each, grown in separate flats, at the same time, in the same growth chamber, under standard (SMB-238 soil, 24°C) conditions. (A) Concentrations of lipid head-groups. Higher-concentration head-groups appear on the left and lower-concentration head-groups appear on the right. (B) Unsaturation in acyl side chain(s). Unsaturation in diacyl lipids (2 acyl chains) appears on the left and unsaturation in lysophospholipids (1 acyl chain) appears on the right. No statistically significant differences between *ala3–*4 and wild-type were observed (p>0.05, Welch’s t-test) either in terms of head-group concentration or unsaturation. Abbreviations: MGDG, monogalactosyldiacylglycerol; PC, phosphatidylcholine; PE, phosphatidylethanolamine; PI, phosphatidylinositol; PA, phosphatidic acid; DGDG, digalactosyldiacylglycerol; PG, phosphatidylglycerol; LPG, lysophosphatidylglycerol; LPC, lysophosphatidylcholine; LPE, lysophosphatidylethanolamine; PS, phosphatidylserine.

To evaluate whether lipid composition is altered in *ala3* pollen, the concentrations of 144 lipids were measured in wild-type and *ala3* pollen grains using tandem mass spectrometry (MS/MS) (Figure 7, File S1). The MS/MS analysis detected polar lipids from 11 different head-groups (MGDG, PC, PE, PI, PA, DGDG, PG, LPG, LPC, LPE and PS) and quantified the acyl carbons and double bonds within the corresponding acyl side chain(s). We chose to examine pollen grains because expression profiling data suggests that ALA3 is preferentially expressed in mature pollen grains and growing pollen tubes (Figure S4) and because the fitness of *ala3* pollen was observed to be temperature-dependent (Table 1). Furthermore, pollen grains could be easily harvested as a pure cell type, minimizing the complications of analyzing tissues made up of different cell types at different developmental stages or physiological states. No differences between *ala3* and wild-type pollen were observed in the concentrations of different head-groups (Figure 7a), or in the amount of double bonds (i.e., unsaturation) within acyl side chains (Figure 7b). These results provide evidence that the concentrations of major membrane-associated lipids in *ala3* pollen are not detectably different from wild-type under standard growth conditions.

## Discussion

Our re-evaluation of *ala3* phenotypes was prompted by the inability of Zhang and Oppenheimer [26] to corroborate a reduced rosette growth phenotype reported by Poulsen et al. [22]. Here we verify the reproducibility of the rosette growth phenotype, and further show that both vegetative (Figures 2 and 3) and reproductive (Table 1) phenotypes are strongly dependent upon growth conditions, including temperature and soil. This indicates that an ALA3 flippase activity is important for growth processes throughout the plant, enabling plants to be more tolerant to varied growth conditions and abiotic stresses.

### The Reduction in *ala3* Rosette Size is Exacerbated by Growth Conditions

A comparison of four standard growth environments revealed a 2-fold difference (40% to 80%) in the average size of the *ala3* rosettes relative to wild-type (Figure 2). The relative reduction in *ala3* rosette size was observed to vary independently with both temperature and soil (Figure S1). All four growth conditions correspond to commonly used, stress-free, growth environments for *A. thaliana*, and included two temperatures (20°C and 24°C) and two commercially available nutrient-rich soils (LB-2 and SMB-238).

It is not clear what differences between the two soils caused the observed variation in *ala3* rosette size. The relatively poor growth of mutants on LB-2 soil was still observed when this soil was supplemented with a “10-10-10 fertilizer” or 1/10 Hoagland’s #2+5 µM Sprint138 chelated iron (data not shown), suggesting that a deficiency in a soil nutrient was unlikely to be the cause of the slow growth. This was further supported by an analysis of the leaf ionome (concentrations of mineral nutrients) of wild-type and *ala3* plants grown under different conditions. No significant differences in the concentrations of 10 elements (Ba, Ca, Fe, K, Mg, Mn, Na, P, S and Zn) (Figure S5) were observed, nor was there any indication of a multi-element profile change that would be diagnostic of a nutritional deficiency for iron or phosphate [30].

### ALA3 is Important for Reproductive Development

The *trans*-Golgi network can function in vesicle trafficking for both secretion and endocytosis [31]. ALA3, which is localized to the *trans*-Golgi network, has been implicated in vesicle budding from this membrane system [22]. To investigate the possible role of ALA3 in a cell type that is dependent on massive vesicle production from the *trans*-Golgi network, we chose to study its contribution to pollen tube growth. The unidirectional growth of pollen tubes is accompanied by very high rates of targeted exocytosis, endocytosis and recycling [32]–[36].

Expression profiling data indicates that ALA3 is primarily expressed in late pollen development and in growing pollen tubes (Figure S4), supporting a role of ALA3 at all stages of pollen maturation and growth. However, in the previous study of ALA3 by Poulsen et al., a version of the ALA3 promoter containing 1,454 bp of the upstream intergenic region was characterized and failed to drive expression of a *GUS*-reporter gene in pollen [22]. An analysis of the intergenic region (3,632 nucleotides) upstream of the ALA3 coding sequence showed the presence of several enhancing and regulatory motifs known to be involved in pollen-specific expression (Figure S6). For example, a motif identical to the tobacco LAT52/56 box (GAAXTTGTGA) is present in the ALA3 intergenic region [37]. Similarly, 18 bp of the ALA3 intergenic region presents an 83% identity to a region of the tobacco LAT52 promoter sufficient to activate pollen-specific transcription [38]. Most of the putative pollen-specific transcriptional enhancers are located upstream of the promoter fragment characterized by Poulsen et al. Although not conclusive, our *in-silico* analysis provides an explanation for the difference between the results obtained in this work and those reported by Poulsen et al.

Zhang and Oppenheimer [26] reported a segregation distortion phenotype for heterozygous *ala3* mutants and provided *in-vitro* evidence of pollen tube growth defects. Our results confirm a segregation distortion phenotype with three independent *T-DNA* insertion alleles (Table 1). In addition, *in-vitro* growth assays indicate that *ala3* pollen tubes germinate with a ∼2 h delay compared to wild-type, and have approximately 2-fold reductions in both maximal growth rate and overall length. These deficiencies explain the 0% success rate of *ala3* pollen in competing with wild-type to fertilize ovules near the bottom of a pistil (Table 2).

The number of seed within individual *ala3* siliques was reduced to 59% that of wild-type (i.e., a 41% reduction). The reduced seed set can be accounted for by a 15–22% reduction in ovule number (Figure 5b) and a high frequency (∼20%) of empty seed positions (Figure 5a, c). While the majority of empty seed positions are located in the bottom of the silique, a weak female transmission deficiency may account for empty positions scattered randomly throughout *ala3* siliques (Figure 5a). Fertilization of *ala3* pistils with wild-type pollen not only reverses the uneven seed set, but also produced siliques with seed counts comparable to the ovule numbers observed in *ala3* pistils (Figure 5b, Figure S3), indicating that, under normal growth conditions, the majority of empty seed positions were caused by a pollen fertilization defect.

At the cellular level, we observed disorganized cytoplasmic streaming in *ala3* pollen. It is not clear what might cause this defect. It is possible that slower processing of vesicles involved in endo- and/or exocytosis causes a vesicular “traffic jam” in pollen tubes. Alternatively, membrane surface features might be altered in such a way that they disrupt or interfere with the dynamic interactions required for coordinating vesicle movement along the cytoskeleton.

### ALA3 is Essential for Hot and Cold Temperature Stress Tolerance

For both root and reproductive development, evidence indicates that ALA3 is important for tolerance to hot- and cold-temperature stresses. The *ala3* root growth phenotype was exacerbated by both hot (30°C) and cold (15°C) temperatures (Figure 3). Interestingly, the mild chilling stress of 15°C was the most harmful to *ala3* root growth, and was eventually lethal to *ala3* seedlings. Similarly, the reduced pollen transmission phenotype was exacerbated (by more than 90-fold) by a hot-day/cold-night stress regime (Table 1). The temperature sensitivity of *ala3* phenotypes suggests a potential parallel with previous reports of cold sensitivity associated with P_4_-ATPase deficiencies in yeast and plants. For example, the yeast Drs2p is required for cell growth at or below 23°C [39], [40]. Similarly, plants with reduced expression of *ALA1* showed a cold-sensitive decrease in plant size [23].

Membrane lipid composition is regarded as being a key factor in temperature stress-tolerance [41]–[48]. The relative concentrations of unsaturated fatty acids [49]–[55], phospholipid head-group classes [56]–[61] and cholesterol [62] have all been linked to low temperature survival in Arabidopsis, tobacco and potato. To address the possibility that *ala3* mutants have an altered lipid composition that might account for their temperature sensitivity, we assayed mature pollen for differences in the concentrations of common head-groups and the levels of unsaturation within fatty acid side chains (Figure 7). However, our analysis failed to reveal any significant differences, indicating that a major global change in the glycerophospholipid composition is not the cause of the defect in *ala3* pollen fitness. A parallel lipid profiling analysis was not done on growing pollen tubes because of the difficulties in obtaining sufficient sample material. Thus, it is still possible that lipid profiles are altered in *ala3* pollen during germination or tube growth. We also cannot exclude the possibility that *ala3* mutants have a more limited change in lipid composition at a specific subcellular location, or the possibility that *ala3* mutants have a reduced ability to rapidly adjust their membrane compositions during a stress response.

Membrane trafficking has also been shown to be linked to multiple stress responses (Reviewed by [63]), including: temperature, salt, osmotic pressure, oxidative conditions, and drought [63]–[69]. It has been hypothesized that vesicular trafficking is essential for the repair of stress-damaged membranes by providing basic “housekeeping” functions, such as the biogenesis, removal and replacement of cellular components [63]. In addition, there are stress response pathways that are specifically linked to membrane trafficking pathways, such as the release of membrane bound transcription factors as part of the ER unfolded protein response pathway [70]. Vesicular trafficking defects have been observed for several P_4_-ATPase mutants in yeast [12], [18]–[21], animals [1], as well as for *ala3* in root columella cells [22].

### Models

While it is not yet clear how a loss of ALA3 can result in the multiple growth-associated phenotypes and temperature sensitivities, two non-exclusive models warrant consideration. First, it is possible that the loss of ALA3 limits the rate at which membrane budding can occur in the *trans*-Golgi, thereby causing a general disruption of the secretory and endocytosis pathways. These pathways are of general importance for all cells, especially pollen tubes that display one of the most rapid polar growth rates of any plant cell. In a second model, the absence of a lipid asymmetry created by ALA3 could impact functional properties, such as membrane fluidity, ion transport, or signaling. For example, a reduced ability to flip PE in *trans*-Golgi vesicles might change lipid/protein interactions that effect enzyme activities [71], or change the availability of PE to function as substrate for the synthesis of other lipid related molecules, such as glycosylphosphatidylinositol anchors [72].

### Conclusions

In summary, we demonstrate that the root, shoot and reproductive phenotypes of *ala3* mutants are strongly dependent upon growth conditions, including soil and temperature. We further demonstrate that *ala3* mutants have decreased fecundity, caused primarily by decreased ovule production and pollen tube growth defects. Together, these results provide evidence that ALA3 functions in multiple cells types and is critical to plant development and adaptation to varied growth environments.

## Materials and Methods

### T-DNA Insertion Mutants and Rescue Lines

Three T-DNA insertional alleles of ALA3 (At1g59820) were used in this study: *ala3-1* (SAIL_422_C12, ss1461), *ala3-2* (SAIL_748_D03, ss1565), and *ala3-4* (SALK_082157, ss836) [73], [74]. All three *ala3* alleles were in the Col-0 wild-type background. The *ala3-1* and *ala3-4* alleles were previously reported by Poulsen et al. [22] and *ala3–4* also corresponds to the *itb2–6* allele used by Zhang and Oppenheimer [26]. Plants harboring *ala3-2* alleles were identified from the SAIL T-DNA collection [74]. The locations of the T-DNA insertions and PCR primers are indicated in Figure 1. PCR primer sequences can be found in File S2. Plant lines expressing the *35s-NTAP2(G)-ALA3* (ps1019) and *35s-ALA3-TAP2(G)* (ps1319) rescue constructs were created as described in Poulsen et al. [22]. Representative transgenic plants rescued by these constructs are ss1252, ss1860 and ss1861.

### Plant Growth Conditions

Unless otherwise stated, seeds were sown on 0.5× Murashige and Skoog (MS) medium (pH 5.7) containing 1% agar and 0.05% MES. Following 48 h of stratification at 4°C, seedlings were grown at room temperature (23°C) under 24 h light for 7–10 d before being transplanted to soil. The soil was Sunshine SMB-238 supplemented with 10-10-10 fertilizer (Hummert) and Marathon pesticide (Hummert) following the manufacturer’s instructions. Plants were grown until maturity in growth chambers (Percival Scientific, Inc., http://www.percival-scientific.com) under a long-day photoperiod (16 h light at 22°C/8 h dark at 20°C, 70% humidity, and ∼125 µmol m^−2^ s^−1^ light intensity).

For experiments to investigate the dependence of *ala3* rosette size under different growth conditions, plants were grown in growth chambers using different combinations of temperature (20°C and 24°C) and soil (SMB-238 and LB-2). The LB-2 soil is a mixture of Canadian sphagnum peat moss, coarse perlite, gypsum, and dolomitic limestone. The SMB-238 soil is a mixture of Canadian sphagnum peat moss, fine perlite, low nutrient charge, gypsum, and dolomitic limestone. In total, four unique combinations of growth conditions were used: (1) 20°C, SMB-238 Soil; (2) 20°C, LB-2 Soil; (3) 24°C, SMB-238 Soil; and (4) 24°C, LB-2 Soil. Upon bolting, the three longest leaves from each plant were collected and photographed using a scanner. Length measurements were made using the ImageJ software package [75].

### Plate-Based Root Growth Assays

Plates were made with 0.5× MS medium, 1% agar, and 0.05% MES and adjusted to pH 5.7 (unless otherwise specified) using KOH. Seeds were stratified for 96 h at 4°C. Seedlings were grown under 24 h fluorescent light (∼100 µmol m^−2^ s^−1^ light intensity) at 26°C unless otherwise specified. Plates were kept at lowered or elevated temperatures using a plate chilling apparatus. Plates were rotated 180° after 3–6 d of plant growth to establish a t = 0 time point. Seedlings were allowed to grow until the longest roots began to reach the bottom of the plate. Plates were photographed using a scanner and length measurements were made using the ImageJ software package [75].

### *in-vitro* Pollen Tube Growth

The pollen tube germination medium (PGM) was based on the method described by Boavida and McCormick [76] and contained: 5 mM CaCl_2_, 0.01% H_3_BO_3_, 5 mM KCl, 10% sucrose, 1 mM MgSO_4_, pH 7.5–7.8, and 1.5% low melting agarose (Nusieve). Pollen from stage 13–14 flowers was applied to its own or a surrogate *ms-1* pistil. Pollinated pistils were placed on ∼400 µL of germination media layered over a microscope slide. The slides were incubated at room temperature (∼23°C) in a petri dish containing water-soaked paper towels to maintain high humidity. Pollen tubes were grown for 2–6 h prior to analysis unless being used for a time course. For time course analyses of pollen tube length, tubes were photographed with a Hamamatsu Orca ER camera attached to a Leica DM-IRE2 microscope using bright-field illumination. Length measurements were done using the ImageJ software package [75].

### Hot-Day/Cold-Night Stress

A growth chamber was used to grow plants under hot-day/cold-night stress conditions (Figure S2). Plants were grown under a long-day photoperiod with temperatures ranging from a peak of 40°C during the day to −1°C at night, with periods of intermediate temperature between the extremes for acclimation. To measure the segregation of *ala3* alleles under hot-day/cold-night stress, plants were first grown under unstressed conditions (see plant growth conditions above) until ∼5 mature siliques were present. The plants were then stripped of immature siliques and open flowers, and moved to the stress chamber where they were grown until senescence. Crosses were performed on plants grown at unstressed temperatures (20–22°C) and manually pollinated plants were immediately moved to hot-day/cold-night stress between 15∶00 and 17∶00 h on the diurnal cycle (chamber temperature of 10°C, Figure S2) and grown until senescence. The progeny of *ala3-1(+/−)* and *ala3-2(+/−)* plants were genotyped by basta resistance and root morphology. A subset of the basta^r^ *ala3-1(+/−)* and *ala3-2(+/−)* progeny were analyzed with PCR to verify the efficacy of genotyping based on root morphology. The progeny of *ala3–4* plants were genotyped using PCR based methods.

### Confocal Microscopy

Images were collected using an Olympus IX81 FV1000 confocal microscope run by the Olympus FluoView 1.07.03.00 software package. A 60× objective (numerical aperture 1.42) was used throughout.

### Progressiveness Ratio

The progressiveness ratio (P) describes the straightness of a trajectory as the ratio of the net displacement between an initial (x_i_, y_i_) and a final (x_f_, y_f_) position, and the total distance covered by all intermediate displacements (∑Dist) [27], [28].

Equation 1:
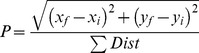



Thus, P = 1.0 for linear movement and decreases as a trajectory becomes less linear.

### Lipid Profiling

Total lipid extracts were obtained from pollen using chloroform/methanol extraction following the protocol provided by the Kansas Lipidomics Research Center (KLRC) (http://www.k-state.edu/lipid/lipidomics/leaf-extraction.html). Lipid extracts were sent to the KLCR for the routine plant polar lipid analysis, in which 144 polar lipids are quantified using precursor and neutral loss electrospray ionization tandem mass spectrometry (ESI-MS/MS).

## Supporting Information

Figure S1 **The size of** ***ala3*** **rosettes relative to wild-type varies with both temperature and soil.** Rosette size was measured at the time of bolting as the average length of the three longest rosette leaves. Rosette sizes were normalized to the wild-type mean and are reported as mean ± SE. Genotypes significantly different from wild-type (p<0.05, Welch’s t-test) appear in gray. Column label abbreviations are as follows: WT represents the wild-type controls; *3-1* and *3-4* represent *ala3-1* and *ala3-4* mutants, respectively; and R represents *ala3* plants rescued by the expression of full length ALA3. Representative results are shown for three independent experiments, n = 7–9 plants for each genotype/condition combination. *Results appearing in Figure 1. ^†^In some cases, *ala3* rosettes were larger than wild-type rosettes. A Mann-Whitney test of all possible *ala3*/WT pairs indicates that the assignment of genotype based on plant size would have been inaccurate 15% of the time. Overlap of *ala3* and wild-type rosette sizes was not observed under any other growth condition.
(PDF)

Figure S2 **Schematic diagram of the hot-day/cold-night temperature stress.** Temperature cycles from 40°C during the day to −1°C at night, with periods of intermediate temperature between the extremes for acclimation. Manually pollinated plants were immediately moved to hot-day/cold-night stress between 15∶00 and 17∶00 h on the diurnal cycle (chamber temperature of 10°C), forcing the period of pollen tube growth and fertilization to overlap with stress temperatures.
(PDF)

Figure S3 **Fertilization of** ***ala3*** **pistils with wild-type pollen resulted in siliques with an even seed distribution.** (A) Representative example of an *ala3* silique fertilized with wild-type pollen. (B) Graph of seed set by quadrant. Siliques were divided into four sectors of equal length, with sector 1 at the top (stigma end) of the silique and sector 4 at the base of the silique. Average results (±SE) are reported for three independent experiments, n = 4–5 siliques. Siliques were collected from three different plants for each *ala3* allele. Sector numbers appear below each column and the average total seed set for each genotype is given above the corresponding sector data.
(PDF)

Figure S4 **Expression profiling data shows preferential expression of ALA3 in mature pollen and growing tubes.** Expression data was obtained from the Arabidopsis eFP Browser (http://bar.utoronto.ca/efp/cgi-bin/efpWeb.cgi) [77] and was normalized against: EF1-alpha (AT5G60390), CBP20 (At5g44200), Actin-2 (At3g18780), and UBC (At5g25760). The lowest normalized expression value (rosette tissue) was arbitrarily set to 1, and the rest of the data adjusted accordingly. Columns representing pollen expression data appear in gray. Expression data for pollen grain maturation [78] and pollen tube growth [79] were collected in independent experiments.
(PDF)

Figure S5 **Elemental concentrations in leaf tissue are not significantly different in** ***ala3*** **and wild-type.** Average results (±SE) for 3–6 independent experiments (n≥20 plants for each genotype) are presented for wild-type (open bars), *ala3-1* (checkered bars), *ala3-4* (gray bars), and *ala3* plants rescued by the expression of full length ALA3 (crosshatched bars). No statistically significant differences between wild-type and any other genotype were observed (p>0.05, Welch’s t-test).
(PDF)

Figure S6 **Several pollen-specific motifs are present in the intergenic region immediately upstream of ALA3.** Sequence data was obtained from The Arabidopsis Information Resource (www.arabidopsis.org) and reads in the 5′ → 3′ direction. Putative conserved regulatory elements were found using the PLACE (A Database of Plant Cis-Acting Regulatory DNA Elements) website (http://www.dna.affrc.go.jp/PLACE/signalscan.html) [80] and the motifs corresponding to the LAT56/59 and the LAT52/56 boxes [37] were searched manually. The sequence used by Poulsen et al. for the ALA3p-GUS analysis [22] appears in bold, underlined text. ORFs for ALA3 (3′ end of sequence) and the immediate upstream gene (5′ end of sequence) appear in gray, uppercase text. Putative regulatory elements are highlighted as follows: Red: sequence similar to the AGAAA TAATAGCTCCACCATA domain of tomato LAT52, where the two underlined motifs are known to form a minimal unit required for pollen-specific expression of the LAT52 promoter. Yellow: enhancing element corresponding to the tobacco LAT52/LAT56 box (GAAXTTGTGA). Green: sequence similar to the tobacco transcriptional enhancer LAT56/LAT59 box element (TGTGGTTATATA). Blue: GTGA motif corresponding to an enhancing element found in the tobacco late pollen gene *g10* and the tomato *LAT56* gene expressed during pollen tube growth.
(PDF)

File S1 **Concentrations of 144 lipids in** ***ala3*** **and wild-type pollen.** This data is summarized in Figure 7. Lipid concentrations were measured using tandem mass spectrometry (MS/MS) that detected 11 different head-groups and quantified the acyl carbons and double bonds within the corresponding acyl side chain(s). Concentrations are expressed as a percentage of the total lipid detected for a specific sample and were calculated using the formula: % total signal = 100 * nmol individual lipid/total nmol for that sample. Pollen was collected from independent groups (n = 4 for WT and n = 3 for *ala3-4*) of ∼75 plants each, grown in separate flats, at the same time, in the same growth chamber, under standard (SMB-238 soil, 24°C) conditions.
(XLSX)

File S2 **PCR primers used to genotype** ***ala3*** **T-DNA insertion lines.** Sequences read in the 5′ → 3′ direction. Lowercase letters in 684a represent introduced restriction sites used for subcloning.
(XLSX)

Movie S1 **Cytoplasmic streaming in wild-type pollen tubes.** Images for this example were taken using DIC microscopy at regular intervals of ∼0.6 s over a 1 m time period. Movie plays at ∼10× speed.
(M4V)

Movie S2 **Cytoplasmic streaming is disorganized in** ***ala3*** **pollen tubes.** Images for this example were taken using DIC microscopy at regular intervals of ∼0.6 s over a 1 m time period. Movie plays at ∼10× speed.
(M4V)
