# Supplementary material for: Loss of the Arabidopsis thaliana P4-ATPase ALA3 Reduces Adaptability to Temperature Stresses and Impairs Vegetative, Pollen, and Ovule Development
Source: PLoS One. 2013 May 7;8(5):e62577. doi: 10.1371/journal.pone.0062577 (PMC3646830; doi:10.1371/journal.pone.0062577)
Supplement: Figure S1 — The size of ala3 rosettes relative to wild-type varies with both temperature and soil. Rosette size was measured at the time of bolting as the average length of the three longest rosette leaves. Rosette sizes were normalized to the wild-type mean and are reported as mean ± SE. Genotypes significantly different from wild-type (p<0.05, Welch’s t-test) appear in gray. Column label abbreviations are as follows: WT represents the wild-type controls; 3-1 and 3-4 represent ala3-1 and ala3-4 mutants, respectively; and R represents ala3 plants rescued by the expression of full length ALA3. Representative results are shown for three independent experiments, n = 7–9 plants for each genotype/condition combination. *Results appearing in Figure 1. †In some cases, ala3 rosettes were larger than wild-type rosettes. A Mann-Whitney test of all possible ala3/WT pairs indicates that the assignment of genotype based on plant size would have been inaccurate 15% of the time. Overlap of ala3 and wild-type rosette sizes was not observed under any other growth condition. (PDF) [file pone.0062577.s001.pdf]

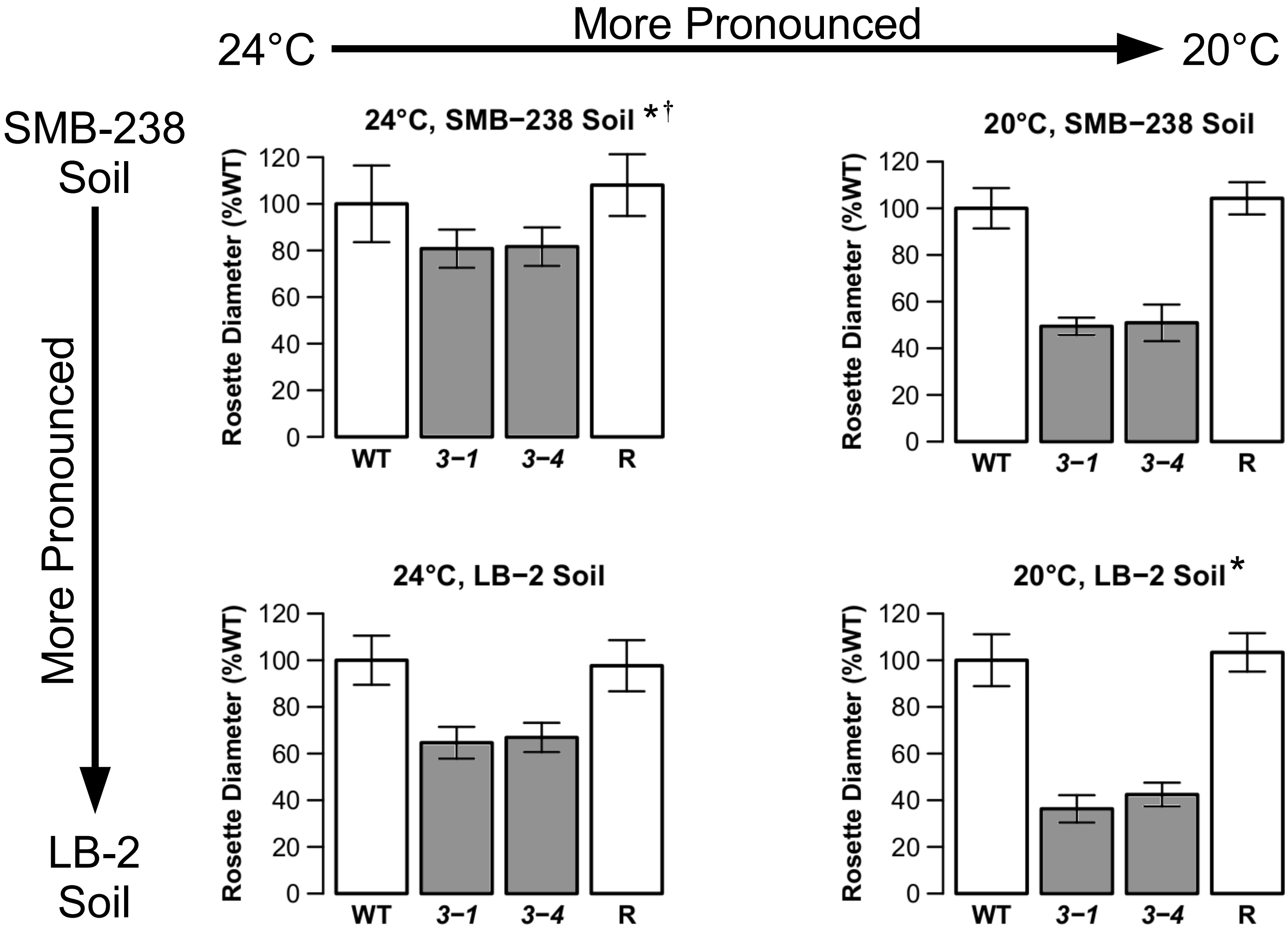

Figure S1. The size of *ala3* rosettes relative to wild-type varies with both temperature and soil. Rosette size was measured at the time of bolting as the average length of the three longest rosette leaves. Rosette sizes were normalized to the wild-type mean and are reported as mean ± SE. Genotypes significantly different from wild-type ($p<0.05$, Welch's t-test) appear in gray. Column label abbreviations are as follows: WT represents the wild-type controls; *3-1* and *3-4* represent *ala3-1* and *ala3-4* mutants, respectively; and R represents *ala3* plants rescued by the expression of full length ALA3. Representative results are shown for three independent experiments, n = 7-9 plants for each genotype/condition combination.

* Results appearing in Figure 1.

† In some cases, *ala3* rosettes were larger than wild-type rosettes. A Mann-Whitney test of all possible *ala3*/WT pairs indicates that the assignment of genotype based on plant size would have been inaccurate 15% of the time. Overlap of *ala3* and wild-type rosette sizes was not observed under any other growth condition.
