# Supplementary material for: Loss of the Arabidopsis thaliana P4-ATPase ALA3 Reduces Adaptability to Temperature Stresses and Impairs Vegetative, Pollen, and Ovule Development
Source: PLoS One. 2013 May 7;8(5):e62577. doi: 10.1371/journal.pone.0062577 (PMC3646830; doi:10.1371/journal.pone.0062577)
Supplement: Figure S2 — Schematic diagram of the hot-day/cold-night temperature stress. Temperature cycles from 40°C during the day to −1°C at night, with periods of intermediate temperature between the extremes for acclimation. Manually pollinated plants were immediately moved to hot-day/cold-night stress between 15∶00 and 17∶00 h on the diurnal cycle (chamber temperature of 10°C), forcing the period of pollen tube growth and fertilization to overlap with stress temperatures. (PDF) [file pone.0062577.s002.pdf]

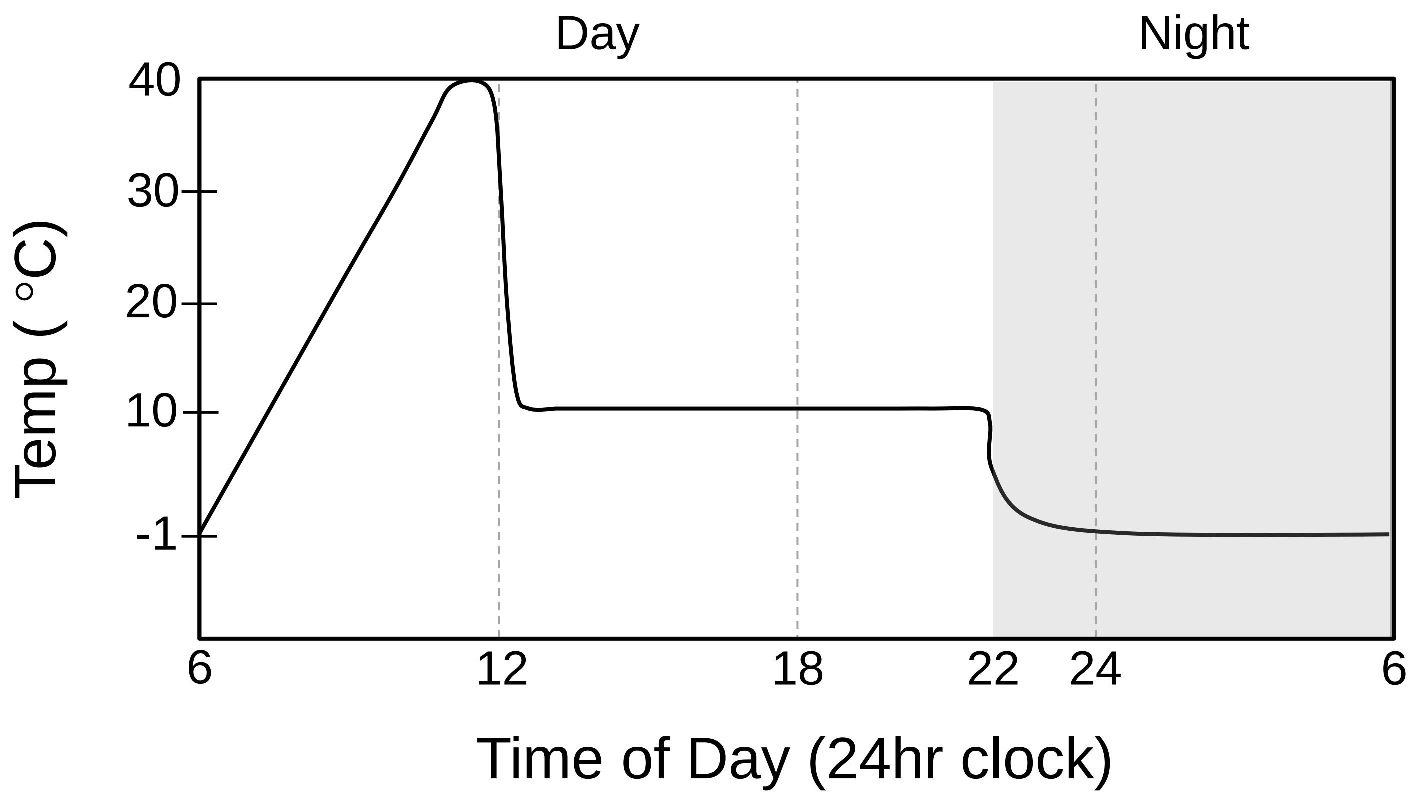

Figure S2. Schematic diagram of the hot-day/cold-night temperature stress. Temperature cycles from 40°C during the day to -1°C at night, with periods of intermediate temperature between the extremes for acclimation. Manually pollinated plants were immediately moved to hot-day/cold-night stress between 15:00 and 17:00 h on the diurnal cycle (chamber temperature of 10°C), forcing the period of pollen tube growth and fertilization to overlap with stress temperatures.
