# Supplementary material for: Loss of the Arabidopsis thaliana P4-ATPase ALA3 Reduces Adaptability to Temperature Stresses and Impairs Vegetative, Pollen, and Ovule Development
Source: PLoS One. 2013 May 7;8(5):e62577. doi: 10.1371/journal.pone.0062577 (PMC3646830; doi:10.1371/journal.pone.0062577)
Supplement: Figure S3 — Fertilization of ala3 pistils with wild-type pollen resulted in siliques with an even seed distribution. (A) Representative example of an ala3 silique fertilized with wild-type pollen. (B) Graph of seed set by quadrant. Siliques were divided into four sectors of equal length, with sector 1 at the top (stigma end) of the silique and sector 4 at the base of the silique. Average results (±SE) are reported for three independent experiments, n = 4–5 siliques. Siliques were collected from three different plants for each ala3 allele. Sector numbers appear below each column and the average total seed set for each genotype is given above the corresponding sector data. (PDF) [file pone.0062577.s003.pdf]

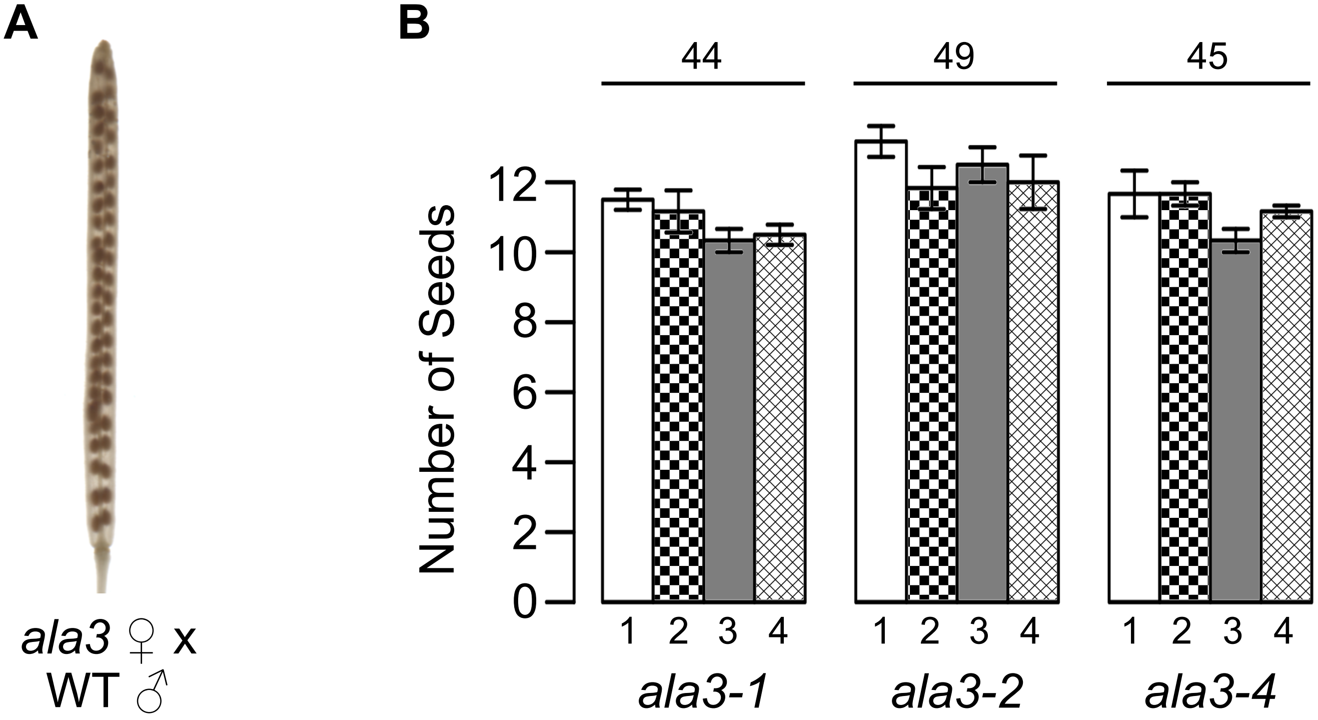

Figure S3. Fertilization of *ala3* pistils with wild-type pollen resulted in siliques with an even seed distribution. (A) Representative example of an *ala3* silique fertilized with wild-type pollen. (B) Graph of seed set by quadrant. Siliques were divided into four sectors of equal length, with sector 1 at the top (stigma end) of the silique and sector 4 at the base of the silique. Average results (±SE) are reported for 3 independent experiments, n = 4-5 siliques. Siliques were collected from three different plants for each *ala3* allele. Sector numbers appear below each column and the average total seed set for each genotype is given above the corresponding sector data.
