# Supplementary material for: Loss of the Arabidopsis thaliana P4-ATPase ALA3 Reduces Adaptability to Temperature Stresses and Impairs Vegetative, Pollen, and Ovule Development
Source: PLoS One. 2013 May 7;8(5):e62577. doi: 10.1371/journal.pone.0062577 (PMC3646830; doi:10.1371/journal.pone.0062577)
Supplement: Figure S4 — Expression profiling data shows preferential expression of ALA3 in mature pollen and growing tubes. Expression data was obtained from the Arabidopsis eFP Browser (http://bar.utoronto.ca/efp/cgi-bin/efpWeb.cgi) [77] and was normalized against: EF1-alpha (AT5G60390), CBP20 (At5g44200), Actin-2 (At3g18780), and UBC (At5g25760). The lowest normalized expression value (rosette tissue) was arbitrarily set to 1, and the rest of the data adjusted accordingly. Columns representing pollen expression data appear in gray. Expression data for pollen grain maturation [78] and pollen tube growth [79] were collected in independent experiments. (PDF) [file pone.0062577.s004.pdf]

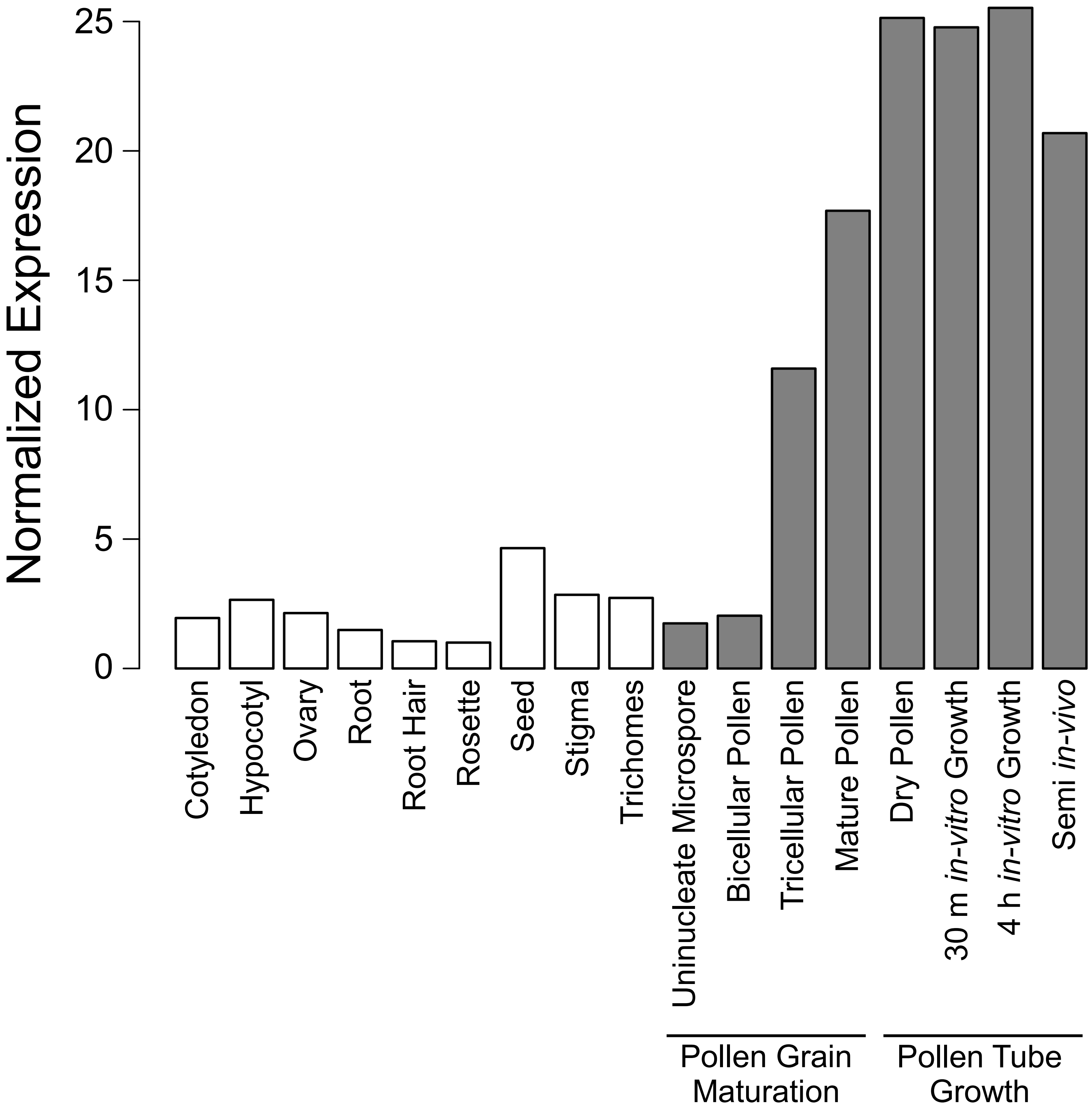

Figure S4. Expression profiling data shows preferential expression of ALA3 in mature pollen and growing tubes. Expression data was obtained from the Arabidopsis eFP Browser (http://bar.utoronto.ca/efp/cgi-bin/efpWeb.cgi) [77] and was normalized against: EF1-alpha (AT5G60390), CBP20 (At5g44200), Actin-2 (At3g18780), and UBC (At5g25760). The lowest normalized expression value (rosette tissue) was arbitrarily set to 1, and the rest of the data adjusted accordingly. Columns representing pollen expression data appear in gray. Expression data for pollen grain maturation [78] and pollen tube growth [79] were collected in independent experiments.
