# Supplementary material for: Loss of the Arabidopsis thaliana P4-ATPase ALA3 Reduces Adaptability to Temperature Stresses and Impairs Vegetative, Pollen, and Ovule Development
Source: PLoS One. 2013 May 7;8(5):e62577. doi: 10.1371/journal.pone.0062577 (PMC3646830; doi:10.1371/journal.pone.0062577)
Supplement: Figure S5 — Elemental concentrations in leaf tissue are not significantly different in ala3 and wild-type. Average results (±SE) for 3–6 independent experiments (n≥20 plants for each genotype) are presented for wild-type (open bars), ala3-1 (checkered bars), ala3-4 (gray bars), and ala3 plants rescued by the expression of full length ALA3 (crosshatched bars). No statistically significant differences between wild-type and any other genotype were observed (p>0.05, Welch’s t-test). (PDF) [file pone.0062577.s005.pdf]

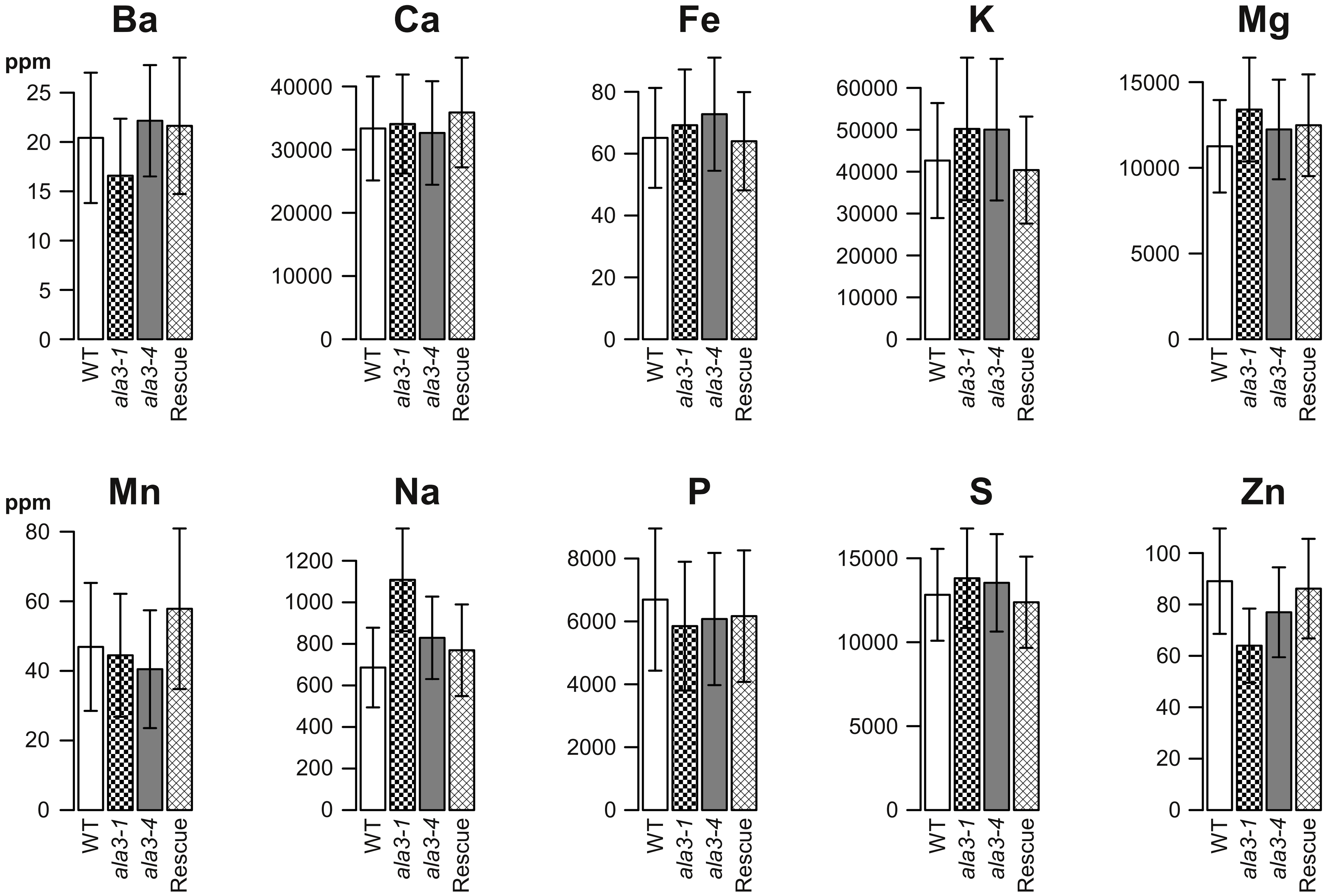


**Figure S5. Elemental concentrations in leaf tissue are not significantly different in *ala3* and wild-type.** Average results (±SE) for 3-6 independent experiments (n ≥ 20 plants for each genotype) are presented for wild-type (open bars), *ala3-1* (checkered bars), *ala3-4* (gray bars) and *ala3* plants rescued by the expression of full length ALA3 (crosshatched bars). No statistically significant differences between wild-type and any other genotype were observed (p > 0.05, Welch's t-test).
