# Supplementary material for: Loss of the Arabidopsis thaliana P4-ATPase ALA3 Reduces Adaptability to Temperature Stresses and Impairs Vegetative, Pollen, and Ovule Development
Source: PLoS One. 2013 May 7;8(5):e62577. doi: 10.1371/journal.pone.0062577 (PMC3646830; doi:10.1371/journal.pone.0062577)
Supplement: Figure S6 — Several pollen-specific motifs are present in the intergenic region immediately upstream of ALA3. Sequence data was obtained from The Arabidopsis Information Resource (www.arabidopsis.org) and reads in the 5′ → 3′ direction. Putative conserved regulatory elements were found using the PLACE (A Database of Plant Cis-Acting Regulatory DNA Elements) website (http://www.dna.affrc.go.jp/PLACE/signalscan.html) [80] and the motifs corresponding to the LAT56/59 and the LAT52/56 boxes [37] were searched manually. The sequence used by Poulsen et al. for the ALA3p-GUS analysis [22] appears in bold, underlined text. ORFs for ALA3 (3′ end of sequence) and the immediate upstream gene (5′ end of sequence) appear in gray, uppercase text. Putative regulatory elements are highlighted as follows: Red: sequence similar to the AGAAA TAATAGCTCCACCATA domain of tomato LAT52, where the two underlined motifs are known to form a minimal unit required for pollen-specific expression of the LAT52 promoter. Yellow: enhancing element corresponding to the tobacco LAT52/LAT56 box (GAAXTTGTGA). Green: sequence similar to the tobacco transcriptional enhancer LAT56/LAT59 box element (TGTGGTTATATA). Blue: GTGA motif corresponding to an enhancing element found in the tobacco late pollen gene g10 and the tomato LAT56 gene expressed during pollen tube growth. (PDF) [file pone.0062577.s006.pdf]

Upstream Gene

TTGCCAAACGATGAGCACCGATATGATTTCAATTTGAtaacttgatgactacgttagtgaaacatgtttctttca
ccttttctcggtgattgatcatttttagtatctaaacttgtatgtttgctccaacacaaatctatttttgtaaagttttggtagtgttttcttgtttattattgatta
atcaaacttttcctttctttctgttacttaaatgctgggcttgatagtagtataagaaatttgtccacaaagcacaccgcaaagttccaaaagctaaaac
ccttgtaccttggagtctctgcatgtgagaaaggaatatgagagttaaaaagagtttttcgtaagacaccaaatcatctacaatgacgatgagcgc
aactttctatcagtgagcttgcaagctagcgttcttgacttcagaaatgtgattatgattaatagttgagttaatcttccttaccaactttaaagaaagaa
aacaatcactatgtcatacattcttcaggggttaaacatgtcggatatgaaagtaaccaaaagaatcctcattgagataagcaagtaataccttcca
acctgcgaaattgttcttgtctggtgattctaaacagaaagaatcttattcttgttttagctcttttgttctagactagacactagtgaaagtgaaatttca

ALA3p-GUS

agaaaggttacaggatcataccttttgtttggtggaaacagagga**ttctgaaattcctggctgtggtcacatacatacatcaatcacatattta**
ctagtttctaatgagaaagttcagcttggattcagttcatcttctctcatctttaaggtccgaactatgtccaaactttggcaatatatttcaa
caaagactctaacattcttatatgtgagtatgtgacgttgtatctcagtaacttgctcatccaactctctgcgaatattcagattcttgattc
aggagctcccaaaacttcggaattccaaatgacgtacacaattgcctaaaacacaagtctcctgcttaaccatgaatagttgagtgaat
caaaatctcactgaacaacgttgtacaagaaatcaaagttcacgatcaaacttacaagcatatccatgaagataaatgagcgaaaact
aattacttggcgtatatatcagttttgacgtttggagctgggaagaaaaatgaaactgcaaatcaaataatgtcaccacattaaattattt
ctgatcgtgtcattattcaactgatagctgttattatttctggtcaacctcttatttgttgtctcttagtaaaagttttcttctagattgaacaat
gttttaaaattttgacaagaagaaaaattaattgtttaccgatttaaagacaagaaagatctaagttagtgtatatttaattaatgcagctt
tagttttttttgttatttggtattttgtcaacaattttttttttataatataatgttcattccacgatcttcggaattgtgcacatccgtcgttcacc
aacataattcaaaggaaccagccaattaaacttagaaaaatattgatgcccatgtcacaacaacgaagaccgttgaaacgacggtgtt
ttacacagccaacgaacaagtccagcatggcggtttttgagaaccaataagaaattaaatgtattgtttactctctagagtaaatttatta
atttttaaagaattcataatattccataaattaataagtttactagcaacaaaaaggtctgaagtctgtatctgacgatcacgcggcctaa
ttgcgcgtggcaatttcttcaacgcggatttttttttcctgccatttcttgcatcaacgcggattaaaaaaaaaaaaaaaagtgttttcggt
aataacccgaaagagaaaatacgtatcggaaaaaatgcaaaattttcaaaaacatttaattttaattttaatttcaagccaaattcggga
gcgactttccaaagacgaacaaagccattgaaacggaagagaaggctgctcctttggtcctctctgtttctaccgtcgattcagatctctg
cctctcgcaatcgaatcaatttgtgtaacgaatcgacgcgaattatagtggtgaagaagtcgacaattcgattgttgtgtaagttgacga

ALA3

gaggattggatcggtgtgaattcctcctaaATGGTTCGATCGGGTAGTTTTAGCGTCGATTCTTCGGCGA
CTCATCAACGGACTCCGTCTCGA

Figure S6: Several pollen-specific motifs are present in the intergenic region immediately upstream of ALA3. Sequence data was obtained from The Arabidopsis Information Resource (www.arabidopsis.org) and reads in the 5’ → 3’ direction. Putative conserved regulatory elements were found using the PLACE (A Database of Plant Cis-Acting Regulatory DNA Elements) website (http://www.dna.affrc.go.jp/PLACE/signalscan.html) [80] and the motifs corresponding to the LAT56/59 and the LAT52/56 boxes [37] were searched manually. The sequence used by Poulsen et al. for the ALA3p-GUS analysis [22] appears in bold, underlined text. ORFs for ALA3 (3’ end of sequence) and the immediate upstream gene (5’ end of sequence) appear in gray, uppercase text. Putative regulatory elements are highlighted as follows:

Red: sequence similar to the AGAAATAATAGCTCCACCATA domain of tomato LAT52, where the two underlined motifs are known to form a minimal unit required for pollen-specific expression of the LAT52 promoter.

Yellow: enhancing element corresponding to the tobacco LAT52/LAT56 box (GAAXTTGTGA).

Green: sequence similar to the tobacco transcriptional enhancer LAT56/LAT59 box element (TGTGGTTATATA).

Blue: GTGA motif corresponding to an enhancing element found in the tobacco late pollen gene *g10* and the tomato *LAT56* gene expressed during pollen tube growth.
